# Draft genome sequence of a Shiga toxin-producing *Escherichia coli* strain from deer meat showing an IS-element integration in the B-subunit of the Shiga toxin Stx2b gene

**DOI:** 10.1128/mra.01093-23

**Published:** 2024-05-03

**Authors:** Michaela Projahn, Sandy Schumann, Sandra Müller, Mathias Ferl, Heike Scholz, André Göhler, Elisabeth Schuh, Maria Borowiak

**Affiliations:** 1 National Reference Laboratory for Escherichia coli including VTEC, Department of Biological Safety, German Federal Institute for Risk Assessment, Berlin, Germany; 2 Department of Food Control, Saxon State Laboratory of Health and Veterinary Affairs, Chemnitz, Germany; 3 Department of Biological Safety, National Study Centre for Sequencing in Risk Assessment, German Federal Institute for Risk Assessment, Berlin, Germany; University of Maryland School of Medicine, Baltimore, Maryland, USA

**Keywords:** *Escherichia coli*, Shiga toxins, transposons, food-borne pathogens

## Abstract

Shiga toxin-producing *Escherichia coli* (STEC) are important food-borne pathogens. Here we report sequence data of the STEC strain BfR-EC-18960, which has integrated IS elements in the B-subunit of the Shiga toxin Stx2b gene. The strain was isolated from deer meat at a local butchery in Germany in 2021.

## ANNOUNCEMENT

Shiga toxin-producing *Escherichia coli* (STEC) are naturally colonizing domestic and wild ruminants (1, 2). The meat of these animals is an important infection source for humans. In Germany, routine food inspection is done by the Federal food laboratories to detect those food contaminations. In this case, STEC strain BfR-EC-18960 was isolated from deer meat through enrichment steps and prescreening real-time PCRs according to ASU L 00.00-150(V) and ISO/TS 13136 (3, 4). Serotyping was done using classical serological testing for the determination of the O-antigen (5) and PCR and RFLP-PCR for H-typing (6) resulting in the serotype O146:H28. Subtyping of the Shiga toxin gene *stx2* revealed an unusual result in the *stx2b*-PCR (6). Furthermore, the RIDASCREEN Verotoxin enzyme immunoassay (r-biopharm AG, Darmstadt, Germany) was negative.

Whole genomic DNA was isolated for short-read Illumina and long-read Oxford Nanopore Technologies (ONT) sequencing as follows. A single colony of strain BfR-EC-18960 was enriched in lysogeny broth for 18–20 h at 37°C. The PureLink DNA Minikit (Invitrogen, Carlsbad, CA, USA) was used for DNA isolation.

The library for short-read sequencing was prepared using the DNA preparation (M) tagmentation kit (Illumina, San Diego, CA, USA). The library was sequenced on the Illumina NextSeq benchtop sequencer using the NextSeq 500/550 midoutput kit v2.5 (300 cycles; Illumina) in 2 × 149 bp cycles. The short-read sequence data were trimmed using fastp v0.19.5 (7). Trimming resulted in 2.291.700 high-quality reads (85% [quality scores of ≥Q30]).

The library for the MinION platform (ONT, Oxford, UK) was prepared using the Rapid Barcoding Kit 96 (SQK-RBK110.96). The MinION library was sequenced for 72 h on an ONT MinION Mk1C device using a MinIon R9.4.1 (FLO-MIN106D) flowcell. Generated fast5 data were basecalled on a high-performance computer using guppy v6.4.8 in SUP mode (https://community.nanoporetech.com/downloads). The obtained reads were trimmed using Porechop v0.2.4 (https://github.com/rrwick/Porechop), filtered using NanoFilt v2.8.0, and quality checked using NanoStat v1.5.0 (8). Trimming and filtering resulted in 55.615 reads with a read N50 value of 9.901  bp.

The DNA isolation and sequencing kits mentioned above were used according to the instructions of the manufacturer. Default parameters were used for all software unless otherwise specified.

**Fig 1:**
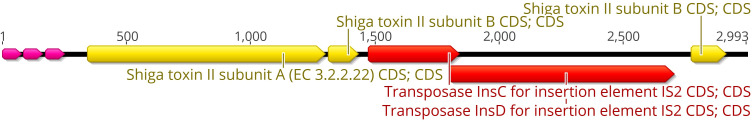
Genomic architecture of the *stx2b* region of STEC strain BfR-EC-18960. Extraction from contig 1 of the unicycler assembly in Geneious Prime 2020.2.2. Colors highlight the annotated tracks by the BV-BRC service: yellow—Stx2b genes, red—insertion elements, pink—tRNAs. Numbers indicate the nucleotide length of the *stx2b* region.

For assembly short- and long-read data sets were subjected to the hybrid assembler Unicycler v0.4.8 including Pilon v1.24 using the bold modus (9–11). In the following, all contigs smaller than 1,000 bp were removed by seqtk1.3 (https://github.com/lh3/seqtk). In the final assembly file, we obtained a set of 15 contigs, with 9 of them being circular. The overall G+C content of the genome sequence was 50.5%. For annotation, the Genome Annotation Service of The Bacterial and Viral Bioinformatics Resource Center (BV-BRC, https://www.bv-brc.org/) was used which is based on the RAST tool kit, RASTtk (12). Analyses of the Stx2b gene region and the IS element insertion (Fig. 1) were done using the Geneious Prime 2020.2.2 software. IS elements are frequently involved in the rearrangement of bacterial genomes (13, 14). The integration into genes encoding Shiga toxin subunits has been reported for individual strains (15, 16) but represents a challenge for routine diagnostics and molecular characterization of isolates from food. Furthermore, IS element integration seems to be not limited to a certain strain or a certain Shiga toxin gene variant.

## Data Availability

Sequencing raw reads were deposited in the NCBI Sequence Read Archive (SRA) (accession numbers SRR26321796 [ONT data] and SRR26321712 [Illumina data]. The draft genome file (SAMN37704675, NZ_JAWPMK010000001.1 to NZ_JAWPMK010000015.1) and the BV-BRC annotated sequence of the stx2b region (Fig. 1, GenBank accession number OR671194) are available at NCBI. The annotation of the draft genome file was added by the NCBI Prokaryotic Genome Annotation Pipeline (PGAP) during upload. All data are encompassed under BioProject accession number PRJNA1024901.
